# Multidrug-resistant conjugative plasmid carrying *mph*A confers increased antimicrobial resistance in *Shigella*

**DOI:** 10.1038/s41598-024-57423-1

**Published:** 2024-03-23

**Authors:** Asaduzzaman Asad, Israt Jahan, Moriam Akter Munni, Ruma Begum, Morium Akter Mukta, Kazi Saif, Shah Nayeem Faruque, Shoma Hayat, Zhahirul Islam

**Affiliations:** Laboratory of Gut-Brain Axis, Infectious Diseases Division (IDD), icddr,b, 68, Shaheed Tajuddin Ahmad Sarani, Mohakhali, Dhaka, 1212 Bangladesh

**Keywords:** Antimicrobials, Bacteria, Clinical microbiology, Drug regulation, Public health, Infectious diseases

## Abstract

Shigellosis remains a common gastrointestinal disease mostly in children < 5 years of age in developing countries. Azithromycin (AZM), a macrolide, is currently the first-line treatment for shigellosis in Bangladesh; ciprofloxacin (CIP) and ceftriaxone (CRO) are also used frequently. We aimed to evaluate the current epidemiology of antimicrobial resistance (AMR) and mechanism(s) of increasing macrolide resistance in *Shigella* in Bangladesh. A total of 2407 clinical isolates of *Shigella* from 2009 to 2016 were studied. Over the study period, *Shigella sonnei* was gradually increasing and become predominant (55%) over *Shigella flexneri* (36%) by 2016. We used CLSI-guided epidemiological cut-off value (ECV) for AZM in *Shigella* to set resistance breakpoints (zone-diameter ≤ 15 mm for *S. flexneri* and ≤ 11 mm for *S. sonnei*). Between 2009 and 2016, AZM resistance increased from 22% to approximately 60%, CIP resistance increased by 40%, and CRO resistance increased from zero to 15%. The *mph*A gene was the key macrolide resistance factor in *Shigella*; a 63MDa conjugative middle-range plasmid was harboring AZM and CRO resistance factors. Our findings show that, especially after 2014, there has been a rapid increase in resistance to the three most effective antibiotics. The rapid spread of macrolide (AZM) resistance genes among *Shigella* are driven by horizontal gene transfer rather than direct lineage.

## Introduction

*Shigella* is the most common pathogen for gastrointestinal infection in developing countries and the leading cause of death among children < 5 years globally^1–5^. *Shigella flexneri* is the predominant strain but *Shigella sonnei* is the uprising strain in low-and-middle-income countries (LMICs) including Bangladesh^5–9^. The sustained pressure of microbial infection and the tendency to quickly reduce the disease duration and severity has led to indiscriminate use of antimicrobials, therefore, triggering the raise of superbugs in developing countries^10,11^. World Health Organization (WHO) recommends ciprofloxacin (CIP) as the first-line therapy along with pivmecillinam, ceftriaxone, and azithromycin as alternative options. Due to the high CIP-resistance in *Shigella* in Bangladesh, the efficacy of CIP is currently in doubt. Recently, *Shigella* isolates have been reported to acquire resistant genes and plasmid with reduce susceptibility to fluoroquinolones and third-generation cephalosporins^12,13^. Ceftriaxone resistance is low in Shigella but it is given parentally, therefore not encouraged for children^14,15^. Therefore, the macrolide AZM is widely used as the most preferred therapy for shigellosis in children^16^.

The IPC policy in Bangladesh is poorly followed and implemented in practice. Indiscriminate use of antibiotics is one of the key aberrant features of antibiotics usage management in LMICs^17,18^. It has been recently reported from Bangladesh that over 25% of the antibiotic users self-medicated themselves and azithromycin was the second highest (21%) self-medicated drug^17,18^.

However, there was no established clinical susceptibility breakpoints of AZM for *Shigella* before 2016^19^. Therefore, in Bangladesh, few studies reported AZM susceptibility for *Shigella* but using different breakpoints which was inconsistent with current CLSI guideline^7,20–23^. Several studies have reported the emergence of AZM resistance in *Shigella spp.* globally^24–26^ and described the mechanism for AZM resistance^23^. To date, different molecular mechanisms involved in the development of resistance to AZM have been described. *Shigella* confers resistance to macrolides through variety of mechanisms include target site modification by methylases, enzymatic inactivation by esterases or phosphotransferases and through efflux pumps^27–30^. Several reports suggested that plasmid-mediated macrolide 2'-phosphotransferase (*mph*A) mostly and esterase (*erm*B) for some instances inactivate macrolide through modifying its molecular structure^31,32^. Furthermore, conjugative R-plasmid mediated horizontal gene transfer (HGT) was demonstrated to be involved in the rapid transfer of genes responsible for resistance^33–36^. In 2015, middle range plasmid (50 MDa) mediated transfer of third generation cephalosporin resistance between *Escherichia coli* and *S. sonnei* was reported in Bangladesh^37^. Recently it has been described that a conjugative R-plasmid carrying azithromycin-resistance genes was involved in reduced susceptibility of *S. flexneri* serotype 3a to AZM^35^.

Furthermore, given the limited treatment options for children with shigellosis, monitoring resistance rates and studying macrolide resistance mechanisms (AZMs) is not only a necessity but a task. Due to the rapid spread of the MDR phenomenon, advanced studies are always needed to assess and track real-time AMR burden in *Shigella*. In this study, we reported a trend towards AMR resistance in *Shigella* and the key mechanism of macrolide resistance in *Shigella* spp.

## Results

### Distribution of *Shigella* strains in Bangladesh between 2009 and 2016

**Figure 1:**
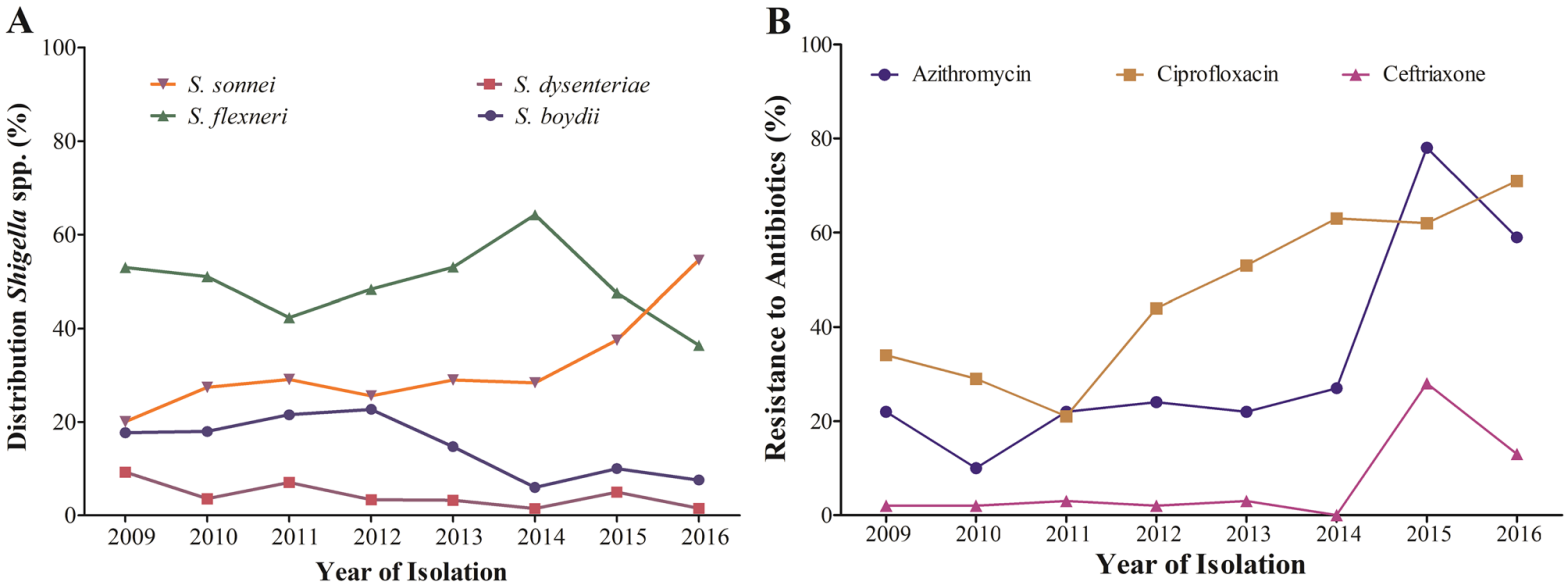
(**A**) Epidemiological distribution of *Shigella* spp. from 2009 to 2016. Different patterns presenting the four species of *Shigella* indicating a clear increasing trend in *S. sonnei* and decreasing trend in *S. boydii* and *S. dysenteriae.* (**B**) Changing pattern in resistance to AZM, CIP and CRO in the time-period of 2009 to 2016. Bar chart indicating an increasing trend for all three most used drugs to treat shigellosis. Rate of resistance showed sharp increase in 2015 and 2016. Resistance to CIP and CRO increasing gradually. Microsoft Excel 2013 was used in visualization.

Distribution of *Shigella* strains (n = 2407) were observed between 2009 and 2016. During this study period, *S. flexneri* was the dominant species (48%) until 2015. However, the prevalence of *S. flexneri* decreased by 17% from 2009 (53%; 314/593) to 2016 (36%; 24/66). At the same time, *S. sonnei* was increased from 20% (119/593) in 2009 to 55% (36/66) in 2016. During this -period, a 10% decrease in the number of *S. boydii* was observed (from 18 to 8%). The frequency of *S. dysenteriae* was consistently low in subsequent years, becoming sporadic (2%) in 2016 (Fig. 1A).

### Susceptibility breakpoints for azithromycin and *Shigella*

**Figure 2:**
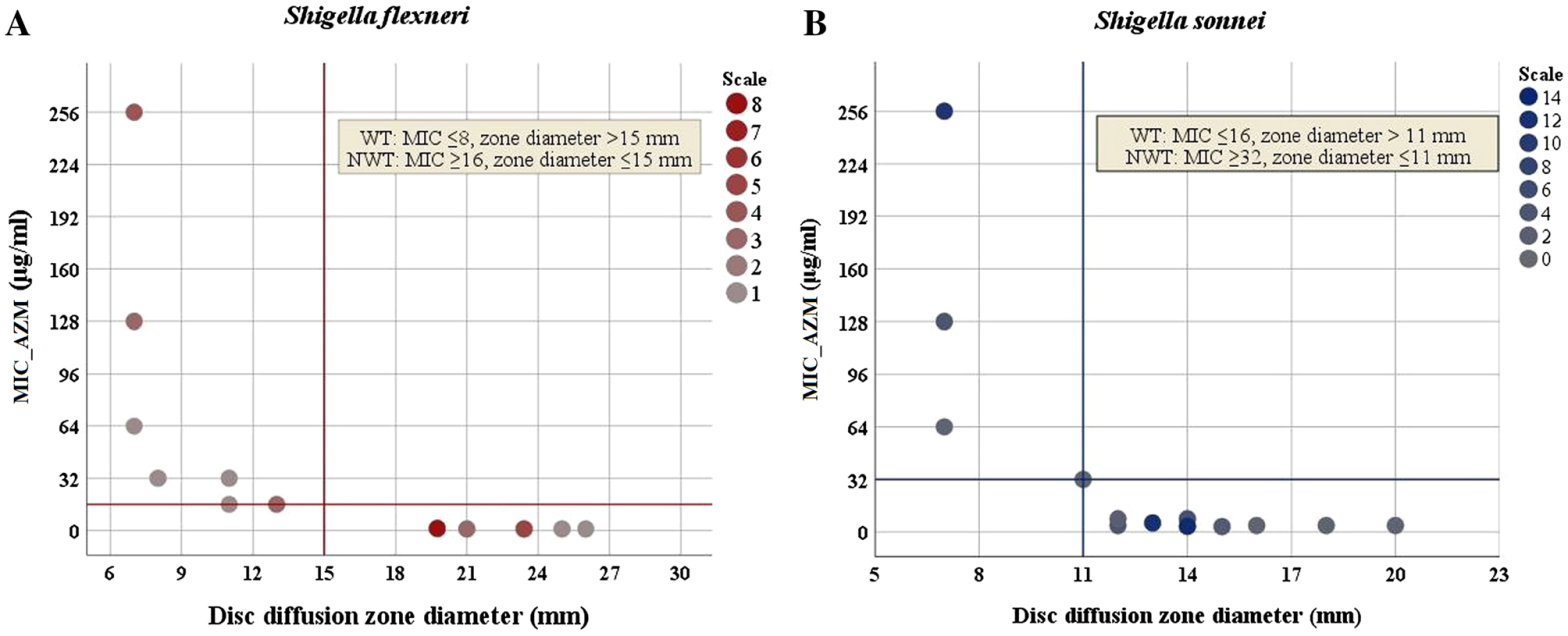
Scatterplot on azithromycin MIC by disc diffusion zone diameter in *Shigella* spp. (**A**) Azithromycin MIC (y axis) and inhibition zone diameter (x axis) showing zone diameter ≤ 15 mm can well segregate susceptible (MIC ≤ 8 µg/ml) and resistant (MIC ≥ 16 µg/ml) in *Shigella flexneri.* (**B**) Azithromycin MIC (y axis) and inhibition zone diameter (x axis) showing zone diameter ≤ 11 mm can well segregate susceptible (MIC ≤ 16 µg/ml) and resistant (MIC ≥ 32 µg/ml) in *Shigella sonnei.* IBM SPSS Statistics 26 were used to generate the figure. AZM = azithromycin, NWT = AZM-resistant, WT = AZM-susceptible, MIC = Minimum inhibitory concentration.

Epidemiological cut-off values (ECVs) are not intended to determine clinical susceptibility cutoffs. Therefore, we performed a non-parametric Spearman rank test between the diameter of the inhibition zone of the azithromycin disk and the available MIC data for 32 *S. flexneri* and 59 *S. sonnei* isolates. A significant correlation was observed between MIC and disc diffusion zone size for both *S. flexneri* (rho, − 0.907; *P* < 0.0001) and *S. sonnei* (rho, − 0.862; *P* < 0.0001). We found no exception to determine the diameter of the disc diffusion zone of *S. flexneri* and *S. sonnei* at the respective MIC values; zone diameter ≤ 15 mm in non wild-type (NWT) *S. flexneri* (MIC ≥ 16 µg/ml) and zone diameter ≤ 11 mm in NWT *S. sonnei* (MIC ≥ 32 µg/ml) (Fig. 2).

### Antibiotic resistance pattern in *Shigella* spp.

A total of 770 *Shigella* strains (336 *S. flexneri*, 233 *S. sonnei*, 162 *S. boydii* and 39 *S. dysenteriae*) were subjected to AST. More than 96% (274/284) of the *Shigella* strains were found resistant to erythromycin and 30% (222/748) to azithromycin (Table 1). In 2014, 27% of Shigella strains were AZM resistant, which was doubled (59%) by 2016 (Fig. 1B). *S. flexneri* and *S. sonnei* were found to confer higher resistance (AZM^R^) than the other two species (Table 1).

**Table 1:** Antibiotic susceptibility of *Shigella* spp. in Bangladesh between 2009 and 2016.

Antibiotics		Number of isolates tested	R/N (%) of Resistant *Shigella spp.*				
			*S. flexneri*	*S. sonnei*	*S. boydii*	*S. dysenteriae*	Total
Macrolide	AZM	748	122/323 (38%)	70/233 (30%)	23/156 (15%)	7/36 (19%)	222/748 (30%)
	ERY	284	109/115 (95%)	105/105 (100%)	52/56 (93%)	8/8 (100%)	274/284 (96%)
Penicillin	AMP	765	195/334 (58%)	44/233 (19%)	62/159 (39%)	19/39 (49%)	320/765 (42%)
	MEL	389	10/185 (5%)	0/67 (0%)	1/106 (1%)	0/31 (0%)	11/289 (3%)
Cephems (Parental)	CRO	595	3/226 (1%)	4/172 (2%)	2/159 (1%)	1/38 (3%)	10/595 (2%)
	CTX	363	34/155 (22%)	5/70 (7%)	2/107 (2%)	0/31 (0%)	41/363 (11%)
	CAZ	331	7/125 (6%)	1/70 (1%)	1/105 (1%)	0/31 (0%)	9/331 (3%)
Cephems (Oral)	CFM	362	40/154 (26%)	4/70 (6%)	4/107 (4%)	1/31 (3%)	49/362 (14%)
Quinolone	CIP	765	149/334 (45%)	178/233 (76%)	9/160 (6%)	1/38 (3%)	337/765 (44%)
	NA	438	136/247 (55%)	119/120 (99%)	25/58 (43%)	7/13 (54%)	287/438 (66%)
FPI	SXT	351	141/248 (57%)	34/42 (81%)	26/51 (51%)	3/10 (30%)	204/351 (58%)

*AMP* ampicillin, *SXT* trimethoprim/Sulfamethoxazole, *NAL* nalidixic acid, *CIP* ciprofloxacin, *CRO* ceftriaxone, *CFM* cefixime, *CTX* cefotaxime, *CAZ* ceftazidime, *MEL* mecillinam, *AZM* Azithromycin, *ERY* erythromycin.

Throughout the study, 44% of the *Shigella* spp. was found CIP-resistant; *S. sonnei* had significantly higher resistance to CIP (76%) compared to *S. flexneri* (45%), *S. boydii* (6%) and *S. dysentery* (3%). In 2016, more than 70% of *Shigella* were found to be resistant to CIP, an increase of 40% since 2009 (30%) (Fig. 1B). Before 2014, CRO resistance was less than 5% but, between 2015 and 2016, CRO resistance increased to 15% (Fig. 1B).

Other third generation cephalosporins including cefotaxime (CTX), ceftazidime (CAZ) and cefixime (CFM) was conferred 11%, 3% and 14% resistance in *Shigella* respectively. In addition, we found 3% resistance to mecillinam (MEL), 42% to ampicillin (AMP) and 58% to trimethoprim-sulfamethoxazole (SXT) in *Shigella.*

### The *mph*A gene conferring decreasing susceptibility to macrolide in *Shigella* spp.

We determined macrolide resistance genes among 37 AZM-resistant *Shigella* spp. which contains 14 *S. flexneri*, 17 *S. sonnei*, 4 *S. boydii* and 2 *S. dysenteriae.* Out of the 37 AZM-resistant *Shigella*, 95% were positive for the *mph*A gene in the PCR test. The remaining 2 isolates did not show a band for any of the macrolide resistance genes studied. The AZM-resistant isolates of *S. sonnei* with a zone diameter ≤ 11 mm (MIC < 32 µg/ml) and AZM-resistant isolates of the other three species with a zone diameter ≤ 12 mm (MIC < 16 µg/ml) in disc diffusion method found positive for the *mph*A gene (Table 2).

**Table 2:** Azithromycin resistance pattern by disc diffusion disc diameter, MIC and presence of gene *mphA.*

AZM disc diffusion zone diameter (mm)	Total no. of isolates	AZM MIC (Ranges in µg/ml)	*S. flexneri*			*S. sonnei*			*S. boydii*			*S. dysenteriae*		
			No. of isolates	MIC (µg/ml)	*mphA*	No. of isolates	MIC (µg/ml)	*mphA*	No. of isolates	MIC (µg/ml)	*mphA*	No. of isolates	MIC (µg/ml)	*mphA*
7 8 9 10 11 12 13 14 15 ≥ 16	28 2 0 1 3 7 13 19 3 25	64–256 32–64 64 4–32 4–16 1–16 1–16 2–4 1–2	8 1 0 0 2 1 1 1 0 18	64–256 32 16–32 16 16 16 1–2	( +)ve ( +)ve ( +)ve (+)ve (−)ve (−)ve ND	16 0 0 0 1 6 12 18 3 3	64–256 32 4–8 2–8 2–8 2–4 4	( +)ve ( +)ve (-)ve ND ND ND ND	3 1 0 0 0 0 0 0 0 5	64–256 64 1	( +)ve ( +)ve	1 0 0 1 0 0 0 0 0 0	256 64	( +)ve ( +)ve

*ND* not done.

### Prevalence of middle-ranged plasmid (MRP) in macrolide resistant strains

**Figure 3:**
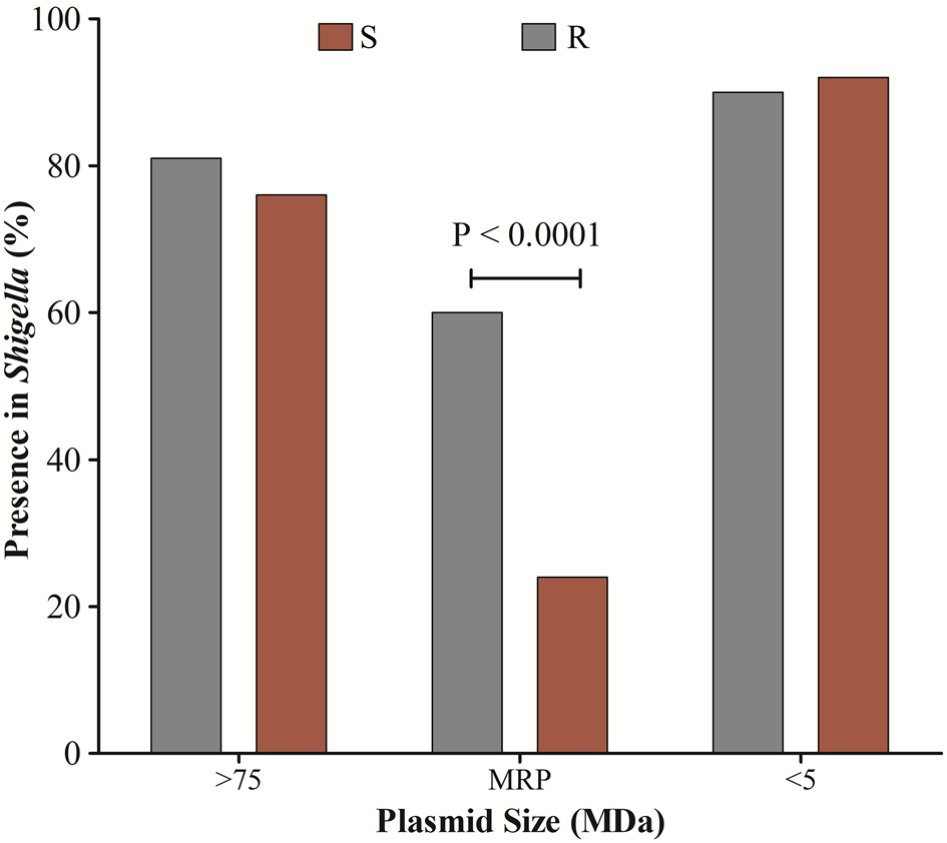
Molecular mechanisms of Macrolide resistance and its dissemination. Distribution of plasmids of different size in macrolide resistant and sensitive strains showing the significantly higher presence of MRP in AZM-resistant *Shigella*. R = AZM-resistant, S = AZM-susceptible, MRP = Middle-range plasmid.

We determined the plasmid profiles of 59 *Shigella* strains; 42 AZM-resistant and 17 AZM- sensitive isolates. Heterogeneous plasmid patterns were distributed in both resistant and susceptible *Shigella* strains. The plasmid size was measured between 1.0 and 140.0 MDa. Almost 80% of the *Shigella* isolates possessed a 140 MDa plasmid and the small plasmid (< 6 MDa) was uniformly distributed in all *Shigella* isolates. Middle-ranged plasmid (MRP) of approximately 35–90 MDa in size was significantly more prevalent (p < 0.0001) in AZM-resistant *Shigella* strains (60%, 25/42) compared to susceptible strains (24%, 4/17) (Fig. 3).

### Horizontal transfer of AMR

**Figure 4:**
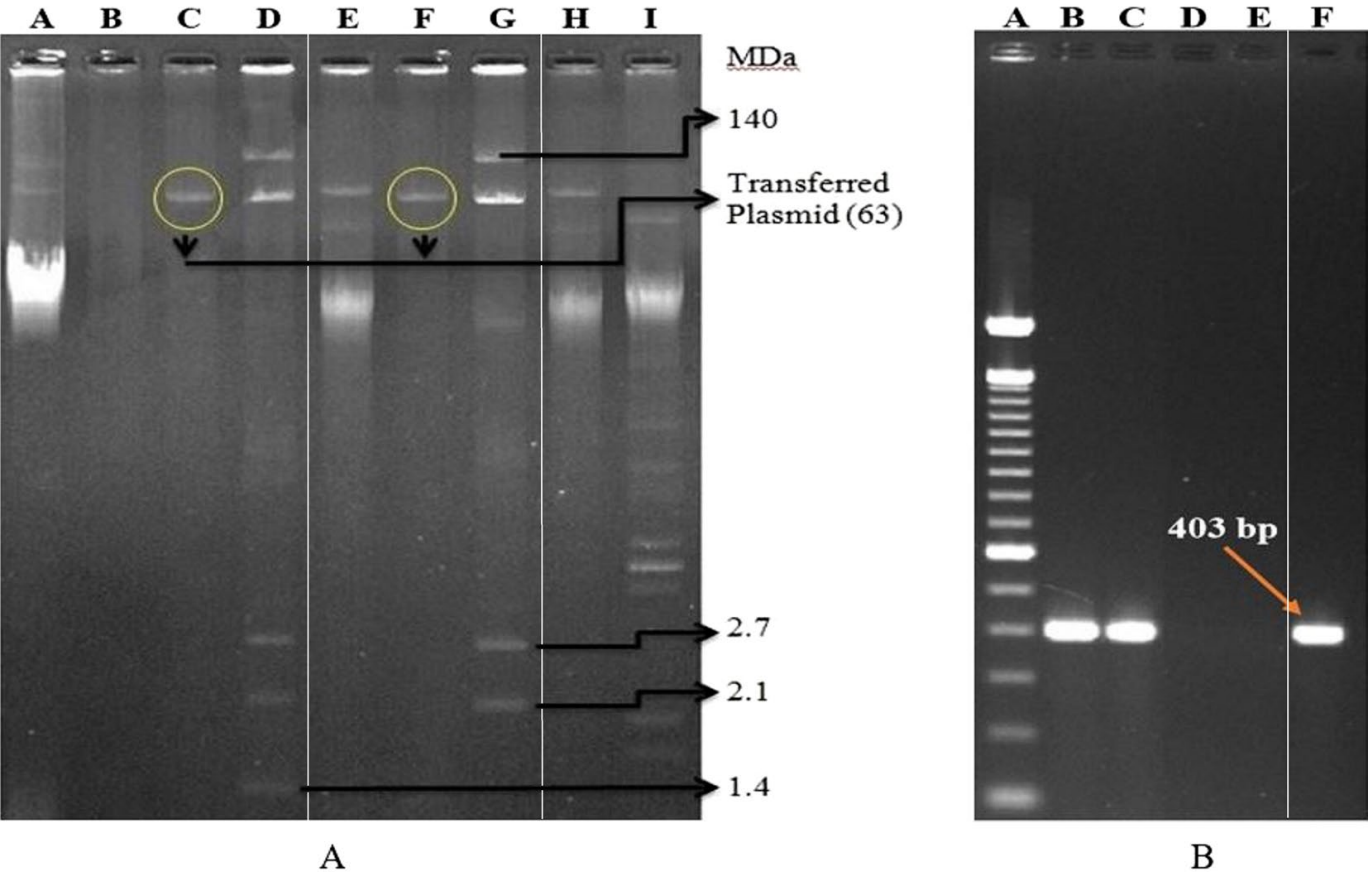
Plasmid and PCR analysis of transconjugants. **(A)** Agarose gel electrophoresis of plasmid DNA from conjugation study showing representative patterns of parent donor strains (Lane D = K12582 and Lane G = K12747), recipient strain (Lane B = K-12), transconjugants (Lane C = Tc-K12582 and Lane F = Tc-K12747) and plasmid size markers (Lane A = PDK-9, Lane E & H = Sa + R1 and Lane I = V-517). (**B)** Gel illusion of mphA gene using plasmid from transconjugant as template. On lane A = Ladder, Lane B = Tc-12582, lane C = Tc-12747, lane D = K-12, lane E = Negative control and lane F = K12747 (PC). *Keynote: Tc = Transconjugant, and PC = Positive control. The original gel images (both A and B) were provided in the Supplementary Fig. S1. Paint application (windows operating system) was used to edit the images.

Antimicrobial susceptibility testing confirmed that the transconjugants resistance to azithromycin, erythromycin, ampicillin and ceftriaxone, same as the donor stains (*S. flexneri* K12582 and *S. sonnei* K12747) (Table 3). The MIC of azithromycin was ≥ 256 µg/ml for all transconjugants. Plasmid analysis of transconjugants revealed that only 63 MDa plasmid was transferred (Fig. 4A) from both donor *Shigella* isolates to *E. coli* K-12 recipient. The *mph*A gene was confirmed in the transconjugants and their plasmid DNAs by PCR (Fig. 4B).

**Table 3:** Transfer of MRP to *E. coli* K-12 during conjugation process.

Strain	Parent Strain			Transconjugant		
	Resistance Pattern	Plasmid profile (MDa)	*mph*A	Resistance pattern	Plasmid profile (MDa)	Resistance factor
*Shigella flexneri* (K12582)	AZM^R^, ERY^R^, AMP^R^, CRO^I^	140, 63, 2.7, 2.1, 1.4	Positive	AZM^R^, ERY^R^, AMP^R^, CRO^I^	63	Positive
*Shigella sonnei* (K12747)	AZM^R^, ERY^R^, AMP^R^, CRO^R^	140, 63, 2.7, 2.1	Positive	AZM^R^, ERY^R^, AMP^R^, CRO^R^	63	Positive

*R* resistant, *AZM* azithromycin, *ERY* erythromycin, *AMP* ampicillin, *CRO* ceftriaxone.

### Plasmid typing and resistome profiling

Analyzing the whole genome sequencing based resistome profiles of the three AZM-resistant *Shigella*, we identified 11, 14 and 15 antimicrobial resistance factors in the genomes of *S. flexneri* Z12966, *S. flexneri* Z13032 and *S. boydii* Z12959 respectively (Table 4). Using PlasmidFinder, we found that all 3 strains were harboring at least a IncFII-type plasmid in their genome. From Mob-suite results, the size of the IncF plasmids were ~ 90 kbp (~ 60 MDa). Separate estimation of AMR-genotype in chromosome and plasmid showed that resistance factors to azithromycin (*mph*A, *erm*B, *msr*E and *mph*E) and cephalosporins (blaTEM-1, blaDHA-1, blaCTXM-15 and blaOXA-1) were plasmid borne, whereas, ciprofloxacin resistance genes were chromosomal (Table 4). In addition, plasmids were conferring the highest and most potential resistance factors in the three *Shigella* genomes studied.

**Table 4:** Resistome profiles of multidrug-resistant *Shigella* spp.

Strain (GenBank Accession)	Plasmid type	^a^AMR genotype	AMR genotype	
			Chromosome	Plasmid
^***b***^***S. flexneri* Z12966 (**JAEUXL000000000**)**	IncFII, Col(MG828), Col(pHAD28), ColpVC	*dfr*A1, *erm*(B), *mph*(A), *mph*(E), *qnr*B4, *sat*2, *bla*EC, *bla*DHA-1, *bla*TEM-1, *msr*(E), *sul*1	*bla*EC, *dfr*A1, *sat*2,	*bla*DHA-1, *bla*TEM-1, *qnr*B4, *sul1*, *mph*(A), *erm*(B), *mph*(E), *msr*(E)
***S. flexneri* Z13032 (**JAFDOL000000000**)**	IncFII	*aad*A1, *dfr*A1, *erm*(B), *gyr*A_S83L, *gyr*A_D87N, *mph*(A), *par*C_S80I, *sat*2, *tet*(B), *bla*EC, *bla*CTX-M-15, *bla*OXA-1, *bla*TEM-1, *cat*A1	*bla*EC, *dfr*A1, *sat*2, *aad*A1, *gyr*A_D87N, *gyr*A_S83L, *par*C_S80I, *cat*A1	*bla*CTX-M-15, *bla*TEM-1, *mph*(A), *erm*(B), *bla*OXA-1, *tet*(B)
***S. boydii* Z12959 (**JAFEJL000000000**)**	IncFII, IncB/O/K/Z, Col(MG828), Col(pHAD28)	*aad*A1, *dfr*A1, *dfr*A5, *erm*(B), *gyr*A_D87Y, *mph*(A), *mph*(E), *qnr*B4, *qnr*S13, *sat*2, *bla*EC, *bla*DHA-1, *bla*TEM-1, *msr*(E), *sul*1	*bla*EC, *bla*DHA-1, *qnr*B4, *gyr*A_D87Y	*dfr*A1, *sat*2, *aad*A1, *bla*TEM-1, *qnr*S13, *mph*(A), *erm*(B), *sul*1, *dfr*A5, *mph*(E), *msr*(E)

^a^Antimicrobial resistance.

^b^*Shigella.*

### Determination of clonal variation of the azithromycin-resistant *Shigella* isolates

**Figure 5:**
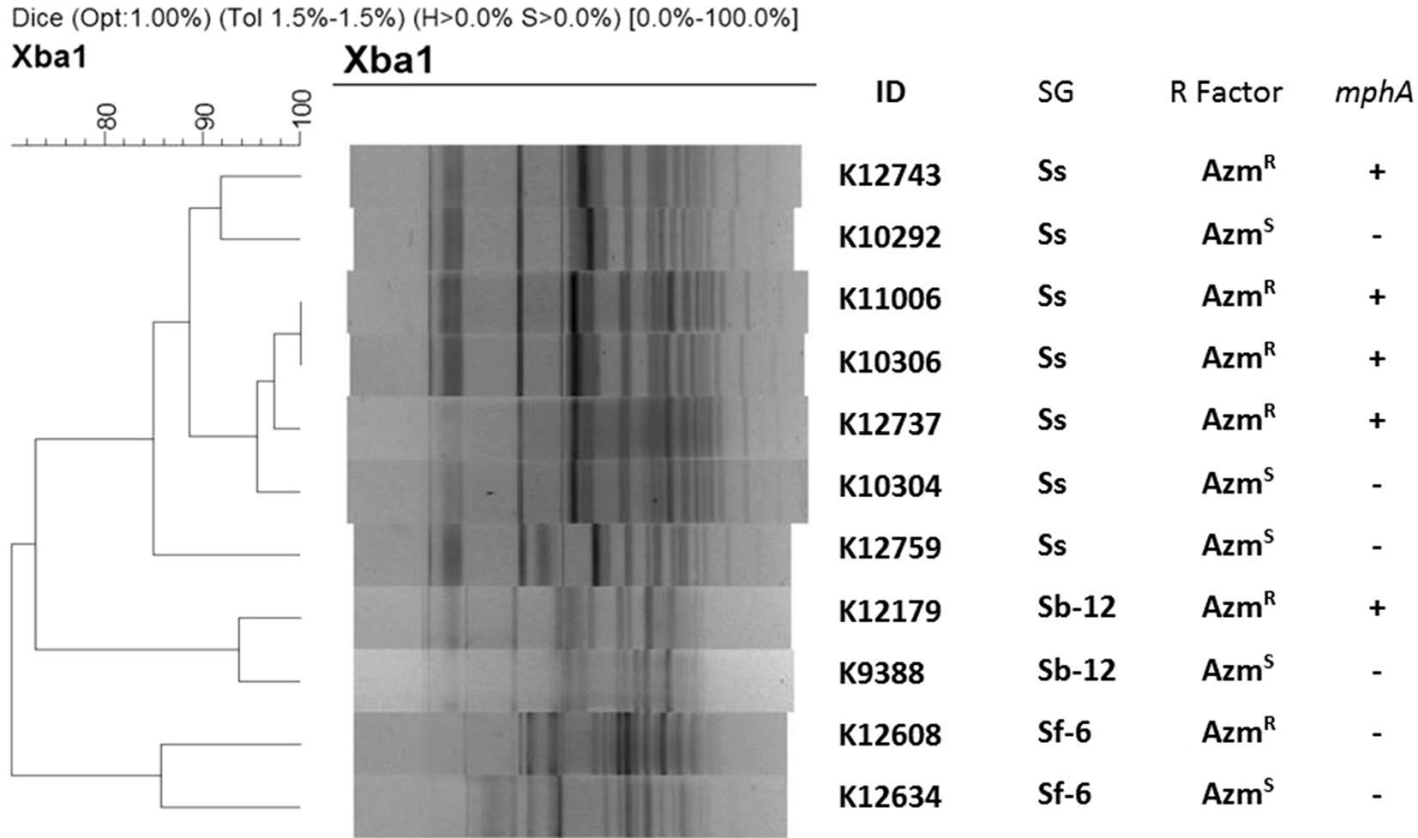
Dendrogram on PFGE gel image containing Xba-I digested chromosomal DNA. Dendrogram generated by BioNumeric software, showing distances calculated by the dice similarity index of PFGE XbaI profiles for AZM-resistant and AZM-sensitive strains. The degree of similarity (%) is shown on the scale.

PFGE analysis of Xba-I digested chromosomal DNA of the Azm^R^ and Azm^S^ *Shigella* strains yielded 21 to 23 reproducible DNA fragments ranging in size approximately from 20 to 690 Kb (Supplementary Fig. S2). Dendrogram on fragment sizes showed no different pulsotype-clustering based on AZM susceptibility. AZM-resistant *Shigella* was not from any single clone of *Shigella* spp. (Similarity < 98%). However, species-wise pulsotype-clustering was present. (Fig. 5).

## Discussion

Antimicrobial resistance has been a long-persistent major public health issue, particularly in underdeveloped and developing nations where shigellosis is endemic. In this study, we report a rapid increase in the resistance to the first-line antibiotics used to treat shigellosis, especially a 40% increase of AZM resistance among *Shigella* spp. in just two years (2014–2016) years. In fact, this is the first report in Bangladesh showing a pattern of AZM-resistance in *Shigella* spp. following the publication of the CSLI defined ECVs for AZM and *Shigella*. The altered temporal dominance of *S. sonnei* over *S. flexneri* has been demonstrated and MRP-mediated HGT is considered to be the main mechanism of AMR spread. Several studies reported emergence of increasing *S. sonnei* worldwide including Bangladesh^6,8^. In 2001, 6% of *S. sonnei* was reported in Bangladesh^6,7,38^, which was increased to 54% in 2016. This acute temporal alteration of *S. sonnei* by 48% in just 15 years seems dramatic in geo-environmental timeframe, gives potential massages of weal and woes in parallel. Continuous improvement in the quality of global drinking water, rapid industrialization, improved nutritional status, better sanitation and less immune-cross-reaction have been resulted in reduced less-adaptive *Shigella* spp. and increased more-adaptive *S. sonnei* load^6,8^. Simultaneously, antibiotic driven immense selection pressure and efficient dissemination channels can resonate the emergence of *S. sonnei* and signs chronic potential problems like spread of MDR *S. sonnei*^39,40^.

A decade ago, several drugs were considered to treat Shigellosis e.g. ciprofloxacin, tetracycline, chloramphenicol, ampicillin, trimethoprim-sulfamethoxazole, nalidixic acid etc^41^. Most of them have long since lost their effectiveness due to low intestinal absorption, cross-reactivity and mainly due to high resistance to *Shigella*.

After being the most preferred treatment option, CIP is seldom prescribed to treat Shigellosis in countries like Bangladesh currently because of its resistance mediated inefficiency^14,42^. In recent years, the prevalence of CIP resistance has been about 70% in patients of all ages, especially in Bangladesh^43,44^. In our study, we also found more than 70% CIP-resistance in 2016. Ceftriaxone is a potential alternative in shigellosis treatment but high cost and route of administration reduces its compatibility^14,15^. Moreover, a rapid increase of CRO-resistance was found in our study.

Empirically administered AZM offers an attractive option for its low frequent dosage system and high intracellular concentration in the colon of patients with shigellosis. The absence of clinical or epidemiological cutoff values lead to unclear conclusions until 2016^45–47^. Previously*, Rahman *et al. followed Antimicrobial Chemotherapy (BSAC) guidelines (sensitive: ≥ 18 mm and resistant: < 18 mm) for AZM breakpoint^22^; *Bourtchai *et al. followed Clinical Laboratory Standards Institute breakpoints recommended for *Streptococci* (> 1 mg/l, resistant; < 0.25 mg/l, susceptible)^20^; *Murray *et al*.* considered all isolates with an MIC of AZM of > 32 isolates as DSA according to CDC^21^. In 2016, CLSI suggested ‘epidemiological cutoff values’ (ECVs)^46^. In 2017, *Darton *et al*.* demonstrated *S. flexneri* (MIC ≥ 16 g/l, zone diameter ≤ 15 mm) and *S. sonnei* (MIC ≥ 32 g/l, zone diameter ≤ 11 mm) breakpoints for AZM based on ECVs of CLSI guidelines^47^. However, there is no clinical breakpoint or ECVs for AZM for *S. boydii* and *S. dysenteriae* in CLSI and EUCAST^46^. Therefore, this confusing situation regarding the AZM breakpoint is not over yet.

In the current study, we found sharp increase of AZM-resistance after 2014 and *mph*A gene was the key mechanism of resistance in molecular and WGS based approaches. Resistome profiling in individual chromosome and plasmid demonstrated the major contribution of plasmid-borne horizontal gene transfer (HGT) in AMR spread rather than clonal expansion. The predominance of HGT over clonality in the spread of azithromycin resistance is reasonable and well addressed in different studies worldwide^35,48–52^. HGT is the most energy-efficient way to transfer genetic material for bacterial species. In addition, HGT allows bacteria inter-species dissemination of AMR factors which is not possible for clonal transfer. A IncFII type plasmid pKSR100 is the most crucial genomic entity fostering AZM resistance^35,52^. We reported for the first time of macrolide resistance pKSR100 when the plasmid carrying IS26–*mph*A*–mrx–mph*R(A)–IS6100 in *Shigella* isolated in Bangladesh the years after 2014^53^.

By correlating the mechanism of AZM resistance, as CRO resistance increased significantly after 2014, the involvement of an emerging R-plasmid carrying CRO-resistant factors in *Shigella* can be strongly inferred. In addition, the transfer of AZM, CRO and AMP resistance phenomena through one conjugative R-plasmid indicate the chance of rapid inter-species dissemination of resistance factors. The involvement of 63 MDA plasmid in AMR-resistance spread well aligns the WGS-based findings and the global studies on the role of IncF plasmids in AMR spread. This size of plasmid is optimum to harbor multiple resistance genes and mechanisms for HGT but can fit into a tiny bacterial genome^54^. We do not think that the pathogen responsible for the acute diarrhea acquires resistance genes immediately upon reaching the hospital as patients with severe diarrhea were admitted to the hospital and fecal samples were collected right after patient’s admission in the hospital. In the PFGE study, the AZM-resistant *Shigella* were not confined in same pulsotype-cluster and they were not from same clone of AZM-resistant bacteria. These findings indicate that horizontal transfer contributes more than direct lineage to spread AMR more rapidly.

Lack of patient data is one of the limitations of the manuscript which might strengthen the schematic illustration of AMR scenario in terms of population features. Although the data presented in the manuscript not from very recent years, it is still appealing and well connected to the current trends of antibiotic resistance in *Shigella* in Bangladesh. Firstly, it fills the information gap by presenting the AMR scenario during the crucial turnover time period of antibiotic resistance in *Shigella* in Bangladesh when the dramatic increase in azithromycin and ceftriaxone resistance happened during the time-period of 2014–2015. Secondly, the data presented in the manuscript bridges the azithromycin resistance rates before and after the epidemiological cut-off value (ECV) of azithromycin for *Shigella* being published in 2016^46^. Lastly but most importantly, azithromycin and third generation cephalosporins (e.g. ceftriaxone) are the most prescribed drugs in case of shigellosis treatment in Bangladesh, therefore, it is crucial to have the clear picture of antibiotic resistance trends.

Overall, the current study provides a clear depiction of the major increase of AMR in *Shigella* and pragmatically evaluates the azithromycin resistance mechanisms in Bangladesh. It signifies the urgency of robust AMR surveillance, resistome profiling and transmission dynamics study in *Shigella* at a large and global scale.

## Materials and methods

### Study population

A total of 2407 *Shigella* strains were identified and isolated between 2009 and 2016 in the Clinical Microbiology Laboratory from the stool specimen of diarrheal patients admitted in icddr,b hospital unit, Dhaka, Bangladesh. Patients with acute watery or bloody diarrhea were kept under observation for two hours upon arriving at the icddr,b hospital. If situation not improve after the observation period providing normal saline and if the watery discharge remains frequent (> 3 times an hour), they were given admission. Only Bangladeshi patients with no history of recent abroad-visit were asked for consent. Fecal samples were collected from diarrheal patients (with consent) and subjected to culture procedures to identify diarrheal pathogen. The identified diarrheal pathogen was subjected to identification tests using phenotypical and *Shigella* spp. specific polyvalent antisera. Diarrheal infection was termed as “Shigellosis” once we confirmed the etiological agent as *Shigella app*. Serotype of all the *Shigella* strains were confirmed in Laboratory of Gut-Brain Signaling, icddr,b, using standard microbiological and biochemical methods^55^. Among these strains, 770 isolates were subjected to antibiotic susceptibility test (AST) and further analysis. This study was reviewed and approved by institutional review board (IRB) of icddr,b, Dhaka, Bangladesh. Three multidrug-resistant *Shigella* (2 *Shigella flexneri* and 1 *Shigella boydii*) isolated in 2017 were used for resistome profiling.

### Serotyping of *Shigella* species

Isolated *Shigella* strains were sub-cultured on MacConkey agar (Difco, Becton Dickinson & Company, Sparks, Md.) plates, and incubated for 16 h for optimum growth. Serotyping was performed by the slide agglutination test^56^. Two types of commercially available kits were used in this study; (i) antisera specific for all type- and group-factor antigens (Denka Seiken, Tokyo, Japan) (ii) monoclonal antibody reagents (Reagensia AB, Stockholm, Sweden) specific for all *S. flexneri* type- and group-factor antigens. After serotyping, single colony of the strains was inoculated in Trypticase soy broth containing 0.3% yeast extract (TSBY), incubated for 16 h and stored at − 70 °C with 15% glycerol afterwards.

### Antibiotic susceptibility tests (AST)

We determined the bacterial susceptibility to antimicrobial agents by the disc diffusion method according to the guidelines of CLSI^46^ using Muller-Hinton agar and commercially available antimicrobial discs (Oxoid, Basingstoke, United Kingdom)^57^. We used *E. coli* (ATCC 25922) strain as negative control in AST. As per CLSI guideline, *S. flexneri* with azithromycin disc diffusion zone diameter ≤ 15 mm and MIC ≥ 16 µg/ml respectively can be considered as NWT. In case of *S. sonnei*, only MIC (WT, ≤ 16 µg/ml and NWT, ≥ 32 µg/ml) was asserted in CLSI guideline^58^. In 2018, Thomas C. *Darton *et al*.* suggested disc diameter ≤ 11 mm as a cutoff value for *S. sonnei*. Thus, we aimed to confirm that disc diffusion zone diameter ≤ 11 mm for *S. sonnei* and ≤ 15 mm for *S. flexneri* can be used to well discriminate AZM-resistant and sensitive isolates in our population. We followed Centers for Disease Control and Prevention (CDC) guided methodology for *S. boydii* and *S. dysenteriae* to define the susceptibility to AZM, where MIC ≥ 32 was defined AZM-resistant (NWT) isolates)^59^. Four different groups of antibiotic discs were used to perform AST: (i) azithromycin (AZM, 15 μg), erythromycin (ERY, 15 μg) from macrolide, (ii) ampicillin (AMP, 10 μg) and amoxicillin/clavulanate (AMC, 10/20 μg) form penicillin, (iii) ciprofloxacin (CIP, 5 μg), and nalidixic acid (NAL, 30 μg) from quinolone and (iv) ceftriaxone (CRO, 30 μg), ceftazidime (CAZ, 30 μg), cefotaxime (CTX, 30 μg) and cefixime (CFM, 30 μg) from cephalosporins and sulfamethoxazole-trimethoprim (SXT, 25 μg). The MIC was determined by the epsilometer test (E-test; AB Biodisk, Solna, Sweden) as per manufacture’s guideline.

### Isolation of plasmid DNA

Plasmid DNA was extracted using modified alkaline lysis method of Kado and Liu^60,61^. Gel electrophoresis was performed in 0.5% agarose gel at 100 V current for 3 h. Gel was stained with ethidium bromide and visualized under ultraviolet rays. The mobility and size of plasmids present in previously described strains *E*. *coli* PDK- 9 (140, 105, 2.7 and 2.1 MDa), R1 (62 MDa), RP- 4 (36 MDa), Sa (23 MDa) and V517 (35.8, 4.8, 3.7, 3.4, 3.1, 2.0, 1.8 and 1.4 MDa) were used as molecular mass standards to scale the unknown plasmid DNA^62^.

### Molecular detection of macrolide resistance genes in *Shigella* species

A total of 37 AZM-resistant *Shigella* isolates for macrolide were selected to extracted the DNA and determine macrolide resistance genes. Polymerase chain reaction (PCR) was performed to determine phosphotransferase genes (*mph*A and *mph*B*),* esterase genes (*ere*A and *ere*B), rRNA methylase genes (*erm*A and *erm*B) and efflux pump mediating factors (*mef*A and *msr*A*)*^63^ (Supplementary Table S1). The primers used for this study were taken from previously published article^20,45^.

### Conjugation and transfer of R-plasmid

We used two multidrug resistant *Shigella* strains as donor strain and *E. coli* K-12 (NAL^R^, Lac^+^, F^-^) as the recipient, to conjugate described previously^64^. In our study, both of the donor strains had an MIC of ≥ 256 µg/ml to azithromycin and were positive for macrolide resistance factor *mphA* gene. Transconjugants were selected on MacConkey agar containing azithromycin (32 μg/ml: Sigma-Aldrich Corporation, St. Louis, Mo.) and nalidixic acid (32 μg/ml: Sigma-Aldrich Corporation, St. Louis, Mo.) that produce lactose-fermenting pink colonies of *E. coli* in contrast to non-lactose-fermenting pale colonies of *Shigella* isolate. As the recipient K-12 was lactose fermenting and susceptible to azithromycin, it can grow only if it receives the AZM resistance factor(s) from donor. The transconjugants were cultured onto MacConkey agar plates, and their identities were reconfirmed as *E. coli* using with API 20E. The selected and confirmed transconjugants were then subjected to plasmid analysis and PCR confirmation for *mphA* gene.

### Resistome profiling

Three MDR-*Shigella* having similar pattern of AMR and conferring middle-range plasmid were subjected to whole genome sequencing using Illumina technologies. Sequence data was processed and assembled using the bio-informatics techniques described previously^57^. Resistome profiling was performed using the AMRFinderPlus v3.10.5^65^ and PlasmidFinder v2.1 (https://cge.cbs.dtu.dk/services/PlasmidFinder/) and MOB-suite v3.1.0 was used to identify plasmids and segregate plasmid-borne AMR genes^66–68^.

### Pulsed-field gel electrophoresis (PFGE)

To observe the clonal relationship between azithromycin resistant and sensitive *Shigella* strains, a total of 11 *Shigella* strains (7 *S. sonnei*, 2 *S. boydii*, 2 *S. flexneri* type 6) were compared using PFGE typing. Genomic DNA of *Shigella* strains was embedded in intact agarose gel and digested for 4 h at 37 °C with XbaI (New England Biolabs) restriction enzyme according to the PulseNet program^69,70^. CHEF-MAPPER system apparatus (Bio-Rad Laboratories) was used to separate the restriction fragments under suitable condition (switching time from 5 to 35 s at 6 V cm^−1^ for 18 h at 14 °C). TIFF image of PFGE were analyzed using BioNumerics version 4.5 (Applied Maths, Kortrijk, Belgium) fingerprinting software. The dendrogram was generated by the UPGMA algorithm with the Dice-predicted similarity value of two PFGE patterns at 1.0% pattern optimization and 1.5% band position tolerance.

### Ethical declaration

All experiments were performed in accordance with relevant guidelines and regulations, and all participants gave their written informed consent prior enrollment. The study was reviewed and approved by the Institutional Review Board (IRB) and the Ethical Committee of icddr,b, Dhaka, Bangladesh.

### Supplementary Information

Supplementary Figure S1.

Supplementary Figure S2.

Supplementary Table S1.

## Data Availability

All data and analysis results generated during this study are included in this article and its supplementary information files; raw data are available from the corresponding author on reasonable request. Sequence data are available under the accession numbers mentioned in the Table 4.
