# Supplementary material for: Multidrug-resistant conjugative plasmid carrying mphA confers increased antimicrobial resistance in Shigella
Source: Sci Rep. 2024 Mar 23;14:6947. doi: 10.1038/s41598-024-57423-1 (PMC10960829; doi:10.1038/s41598-024-57423-1)
Supplement: Supplementary file 1 — Supplementary Figure S1. [file 41598_2024_57423_MOESM1_ESM.pdf]

Multidrug-resistant conjugative plasmid carrying *mph*A confers increased antimicrobial resistance in *Shigella*

Supplementary figure S1

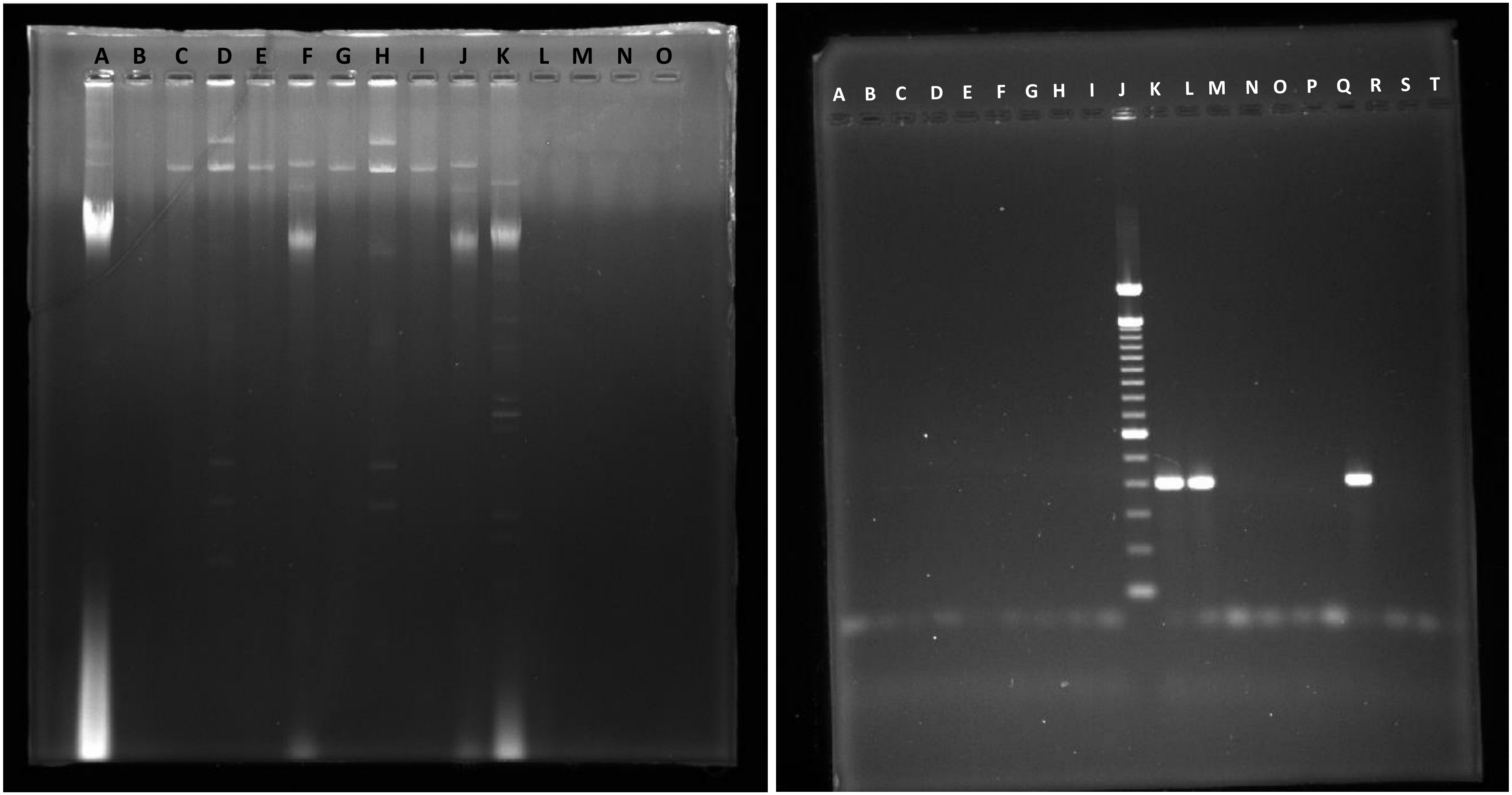

A B

Supplementary figure S1: Plasmid and PCR analysis of transconjugants. **(A)** Agarose gel electrophoresis of plasmid DNA from conjugation study showing representative patterns of parent donor strains (Lane D = K12582 and Lane H = K12747), recipient strain (Lane B = *E. coli* K-12), transconjugants (Lane C & E = Tc-K12582 and Lane G & I = Tc-K12747), plasmid size markers (Lane A = PDK-9, Lane F & J = Sa+R1 and Lane K = V-517) and Blank (Lane L-O). **(B)** Gel illusion of mphA gene using plasmid from transconjugant as template. On lane A-I & R-T = Regular laboratory samples, lane J = 100 bp Ladder, Lane K = Tc-12582, lane L = Tc-12747, lane M = K-12, lane N = Negative control, lane O = Reagent blank, lane P = Blank, lane Q = K12747 (PC). *Keynote: Tc = Transconjugant, and PC = Positive control. Paint application (windows operating system) were used to edit the images.
