# Supplementary material for: Multidrug-resistant conjugative plasmid carrying mphA confers increased antimicrobial resistance in Shigella
Source: Sci Rep. 2024 Mar 23;14:6947. doi: 10.1038/s41598-024-57423-1 (PMC10960829; doi:10.1038/s41598-024-57423-1)
Supplement: Supplementary file 2 — Supplementary Figure S2. [file 41598_2024_57423_MOESM2_ESM.pdf]

Multidrug-resistant conjugative plasmid carrying *mph*A confers increased antimicrobial resistance in *Shigella*

Supplementary figure S2

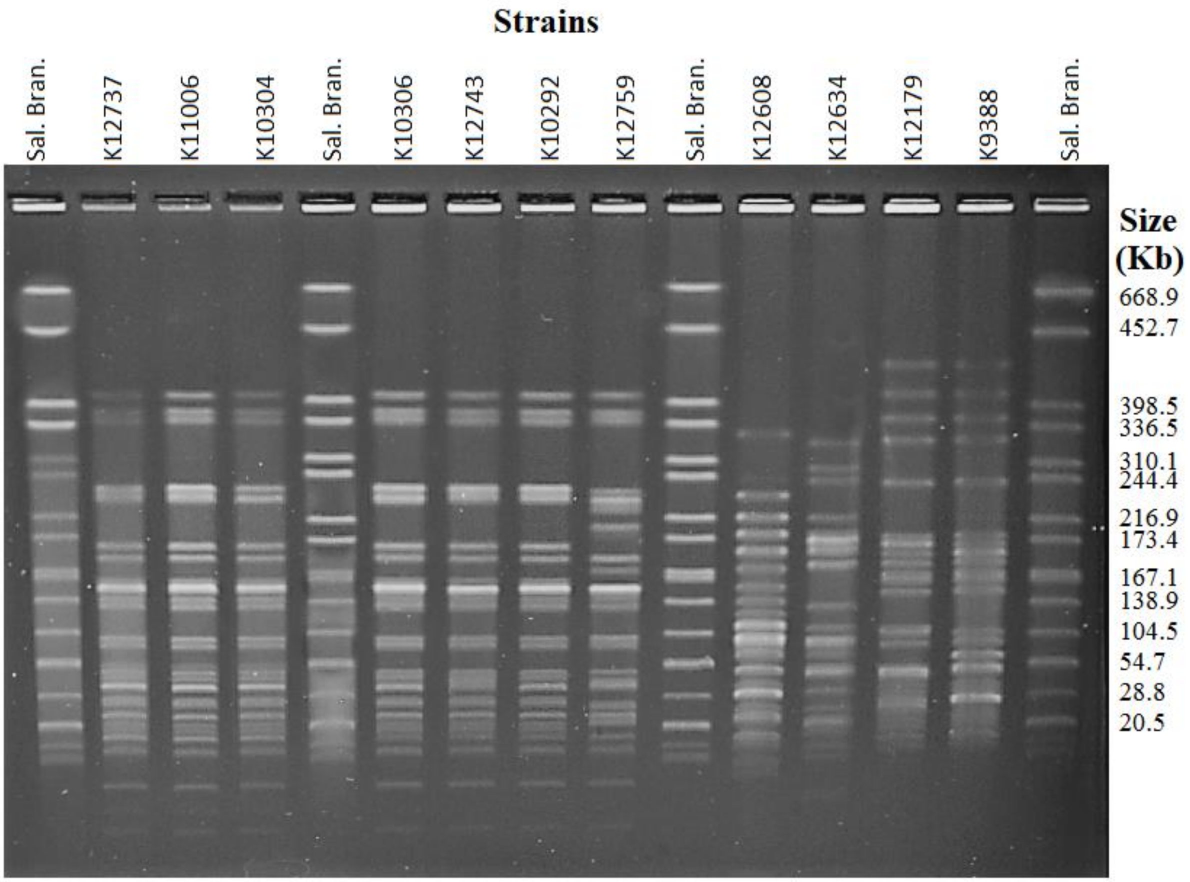

Supplementary figure S2: PFGE Banding Patterns of *Xba*I-digested Chromosomal DNA of Representative Isolates of Azm^{R} (K12737, K11006, K10306, K12743, K12608, K12179) and sensitive (K10304, K10292, K12759, K12634, K9388) *Shigella* isolates. Paint application (windows operating system) were used to edit the images.
