# Supplementary material for: Multidrug-resistant conjugative plasmid carrying mphA confers increased antimicrobial resistance in Shigella
Source: Sci Rep. 2024 Mar 23;14:6947. doi: 10.1038/s41598-024-57423-1 (PMC10960829; doi:10.1038/s41598-024-57423-1)
Supplement: Supplementary file 3 — Supplementary Table S1. [file 41598_2024_57423_MOESM3_ESM.docx]

**Multidrug‑resistant conjugative plasmid carrying *mph*A confers increased antimicrobial resistance in *Shigella***

**Supplementary table S1:**

| Supplementary table S1. Oligonucleotide primers used for the detection of macrolide resistance genes by PCR | | | | | |
| --- | --- | --- | --- | --- | --- |
| Function | Target gene | Primer | Sequence, 5’→3’ | Product Size, bp | Annealing temperature (°C) |
| Macrolide Phosphorylation | *mphA* | Forward  Reverse | GTGAGGAGGAGCTTCGCGAG  TGCCGCAGGACTCGGAGGTC | 403 | 60 |
|  | *mphB* | Forward  Reverse | GATATTAAACAAGTAATCAGAATAG  GCTCTTACTGCATCCATACG | 494 | 58 |
| Methylation | *ermA* | Forward  Reverse | TCTAAAAAGCATGTAAAAGAAA  CGATACTTTTTGTAGTCCTTC | 533 | 52 |
|  | *ermB* | Forward  Reverse | GAAAAAGTACTCAACCAAATA  AATTTAAGTACCGTTACT | 639 | 45 |
|  | *ermC* | Forward  Reverse | TCAAAACATAATATAGATAAA  GCTAATATTGTTTAAATCGTCAAT | 642 | 45 |
| Esterase | *ereA* | Forward  Reverse | GCCGGTGCTCATGAACTTGAG  CGACTCTATTCGATCAGAGGC | 420 | 60 |
|  | *ereB* | ereBF  ereBR | TTGGAGATACCCAGATTGTAG  GAGCCATAGCTTCAACGC | 537 | 55 |
| Efflux Pump | *mefA* | mefAF  mefAR | AGTATCATTAATCACTAGTGC  TTCTTCTGGTACTAAAAGTGG | 345 | 54 |
|  | *msrA* | msrAF  msrAR | GCACTTATTGGGGGTAATGG  GTCTATAAGTGCTCTATCGTG | 384 | 58 |
